# Time‐varying flow–ecology relationships for an endangered fish population: Longfin Smelt in the San Francisco Estuary

**DOI:** 10.1002/eap.70178

**Published:** 2026-01-19

**Authors:** Parsa Saffarinia, James A. Hobbs, Stephanie M. Carlson, Albert Ruhí

**Affiliations:** ^1^ Department of Biological Sciences California State University Chico California USA; ^2^ Department of Environmental Science, Policy, and Management University of California, Berkeley Berkeley California USA; ^3^ California Department of Fish and Wildlife, Bay‐Delta IEP Stockton California USA; ^4^ Department of Wildlife, Fish and Conservation Biology University of California, Davis Davis California USA

**Keywords:** biomonitoring, estuarine ecosystems, non‐stationarity, river flow regime, time‐series analyses

## Abstract

Major estuaries globally are experiencing fast‐paced changes in hydrology and ecosystem dynamics. However, connecting alteration of river flow regimes to estuarine fish population dynamics remains a challenge, partly due to the untested assumption that flow regimes, fish dynamics, and the resulting flow–ecology relationships are stationary (i.e., have no systematic changes in mean or variance over time). Here, we studied the endangered population segment of Longfin Smelt (*Spirinchus thaleichthys*) in the San Francisco Estuary, which depends on seasonal river flows to reproduce. We used extensive biomonitoring data (1980–2020) and two time‐series modeling techniques, namely multivariate autoregressive state‐space (MARSS) models and dynamic linear models (DLMs), to understand how population dynamics respond to interannual flow variation, and whether flow–ecology relationships have changed over time. MARSS outputs showed that population trajectories are best explained by a combination of lateral and vertical dimensions of habitat structure, that is, whether individuals were collected in channels versus shoals, and in pelagic versus benthic environments. In turn, DLMs revealed time‐varying, but often positive effects of flow on young‐of‐the‐year abundance in shallow channel and shoal habitats, but no consistent relationships for older individuals (age‐1+), likely due to other drivers influencing survival from age‐0 and age‐1+. Finally, we found that the two modeling approaches showed agreement only in about 30% of the cases. Divergence in the sign and/or magnitude of flow effects suggests that time‐averaged approaches may sometimes oversimplify non‐stationary relationships between the environment and fish population dynamics. From a conservation standpoint, the gradually weakening but positive flow–ecology relationship (as opposed to a step change in the relationship) suggests that it may still be possible to reverse the steep population declines of Longfin Smelt through a combination of flow and habitat restoration actions. While we focused on a particular endangered population, our quantitative approach is transferable to other taxa and geographies, and could help inform management of flow‐dependent resources in systems strongly affected by non‐stationarity. We contend that time‐varying flow–ecology relationships are needed to better capture ecological realism, and could help design more effective conservation strategies in fast‐changing environments.

## INTRODUCTION

River flows have a profound influence on estuarine habitats and biota, creating dynamic salinity gradients that freshen otherwise brackish habitats at tidal, seasonal, and interannual timescales (Chilton et al., 2021; Vale & Dias, 2011). River flow regimes, or the characteristic patterns of flow variation over time (Poff et al., 1997), are known to control ecological dynamics in watersheds, from headwaters to river mouths (Palmer & Ruhí, 2019). These flow–ecology relationships have been well documented at multiple levels of biological organization, including in estuaries. For example, changes in salinity, turbidity, and flow velocity influence population dynamics in a wide range of estuarine fishes, including the federally endangered Delta Smelt in California (Bever et al., 2016). At the community level, flow variation often produces distinct fish assemblages during wet relative to dry periods (Vorwerk et al., 2008) and influences behavior and recruitment of anadromous and estuarine opportunists (Colombano et al., 2022). At the food web level, low river outflows can reduce connectivity across estuarine regions (Vinagre et al., 2011) and decrease tidal marsh primary productivity (Parker et al., 2011). Such relationships between aspects of the flow regime and key ecosystem processes have motivated the field of environmental flow science, which aims to balance flows for ecosystem needs with societal demands for freshwater. Central to this tenet is the assumption that flow–ecology relationships are robust and static. Despite calls to challenge this assumption (Chen & Olden, 2018; Poff, 2018; Tonkin et al., 2019), stationarity in flow–ecology relationships is rarely tested.

Many aquatic systems have undergone fast‐paced ecological change due to human appropriation of river flows, invasive species, declines of native species, and climate change (Biguino et al., 2023; Cloern et al., 2016). In estuaries, these combined stressors have often led to abrupt ecosystem change (Cloern & Jassby, 2012). Thus, understanding to what extent historical flow–ecology relationships remain valid in heavily impacted systems has far‐reaching implications. For example, using flow–ecology relationships, flows of the right magnitudes and at the right times have been found to mitigate the effects of invasive species (Gillson, 2011; Ibanez et al., 2020). Similarly, flow–ecology relationships in the Yangtze Estuary have informed optimal flow ranges to aid in the management of native fishes (Huang et al., 2024). Understanding whether and how flow–ecology relationships may be changing is thus a pressing need with management implications in aquatic ecosystems around the globe (Poff, 2018).

Quantitative approaches based on time‐series analysis have been useful for identifying flow–ecology relationships, partly due to their ability to disentangle flow effects from effects of the population (or “process”) on itself. Time‐series approaches have traditionally assumed static flow–ecology relationships, but new tools are emerging that challenge assumptions of stationarity—that is, the absence of systematic changes in mean, variance, or periodicity. For example, Ruhi et al. (2018) explored the potential for time‐varying flow–ecology relationships using wavelets and multivariate autoregressive (MAR) models that described the transient effects of flow management for hydropower operations on the functional structure of aquatic invertebrate communities. Similarly, Paudel et al. (2020) examined dynamics of endangered river dolphins via generalized additive models and showed that time‐varying flow induced large biological responses across a spatiotemporal gradient, along which taxa adjusted their life histories to persist. Dynamic linear models (DLMs) have also been used to explore time‐varying relationships within different facets of the environment, or between environmental drivers and ecological responses. For instance, Leathers et al. (2023) recently linked time‐varying thermal sensitivity to vulnerability of riverine invertebrates exposed to drought. In short, there is a small but growing body of work exploring—and documenting—shifting flow–ecology relationships in a wide range of aquatic ecosystems.

The San Francisco Estuary‐Bay Delta system (hereafter, the Estuary) supports high levels of ecological diversity and productivity, despite being one of the most heavily impacted and managed ecosystems in the world (Cloern & Jassby, 2012; Nichols et al., 1986). The Estuary is characterized by strong seasonal and interannual variability in hydrology (Hutton et al., 2017), with river outflows supporting agricultural and environmental water needs (Lund, 2016), including freshwater habitat for imperiled fishes (Kimmerer, 2002a, 2002b) and birds. Notably, the ecosystem has been changing rapidly due to multiple global change stressors (Bashevkin et al., 2022; Stern et al., 2020). For example, recent multiyear droughts have altered important environmental conditions such as temperature, salinity, turbidity, and nutrient availability, with implications for endangered fishes (Bosworth et al., 2024; Hartman et al., 2024). Additionally, the spread of non‐native species has fundamentally disrupted food‐web structure by sequestering energy from pelagic to benthic habitats (Cloern & Jassby, 2012; Kimmerer et al., 1994; Winder & Jassby, 2011). These changes in environmental conditions and food availability have led to steep declines of native pelagic fishes, and a renewed focus on management informed by flow–ecology relationships. For example, juvenile Chinook Salmon (*Oncorhynchus tshawytscha*) entrainment probability at water export facilities in the Bay‐Delta ecosystem depends on inflows of freshwater and tide stage (Perry et al., 2015), and flow‐mediated turbidity initiates the spawning migration of the endangered Delta Smelt (*Hypomesus transpacificus*) (Bennett & Burau, 2015). Altered flows have also been shown to significantly alter the distribution of several estuarine forage fishes (Grimaldo et al., 2020). Interannual variation in flow continues to influence persistence for many estuarine fishes (Edgar et al., 2000; Feyrer et al., 2015; Tamburello et al., 2019), as well as the energy pathways that support them (Pagliaro et al., 2025).

Longfin Smelt (*Spirinchus thaleichthys*) have been declining for several decades in the San Francisco Estuary (Rosenfield & Baxter, 2007; Sommer et al., 2007; Tobias et al., 2023), which prompted this population segment to be recently listed as endangered under the United States Endangered Species Act (U.S. Fish and Wildlife Service, 2024). Several factors are assumed to have contributed to Longfin Smelt declines, including flow‐mediated increases in salinity (i.e., reduced availability of low‐salinity zones), decreased turbidity, high water temperatures, and food limitation (Hobbs et al., 2017; Jeffries et al., 2016; Rosenfield & Baxter, 2007). Longfin Smelt populations have been at critically low levels in the Estuary since around 2000, coinciding with the onset of the Pelagic Organism Decline (POD; Sommer et al., 2007), which suggests that ecosystem regime shifts may have contributed to their decline (MacNally et al., 2010; Thomson et al., 2010). Importantly, as Longfin Smelt populations have declined to low levels, the drivers of their dynamics may also be changing—including the magnitude of their responses to freshwater outflows. Available long‐term hydrological and biomonitoring data provide an opportunity to explore this possibility (Tempel et al., 2021; Young et al., 2024) and inform potential pathways to recovery.

In this paper, we linked interannual variation in estuarine river outflows to Longfin Smelt population dynamics via advanced time‐series methods. We aimed to: (1) determine whether Longfin Smelt population fluctuations have been spatially consistent across the Estuary; (2) quantify the effects of freshwater outflow on Longfin Smelt populations; (3) determine whether the outflow–Longfin Smelt abundance relationships have changed in magnitude or sign over time; and (4) compare outputs of two complementary time‐series methods to determine whether allowing flow effects to be time‐varying changes our understanding of Longfin Smelt dynamics and its drivers.

We hypothesized that (1) habitat type, structured over lateral and vertical gradients (i.e., channel vs. shoal, and pelagic vs. benthic environments, respectively) would play a key role in defining Longfin Smelt population trends, since turbidity has been decreasing in the Estuary, thereby increasing vulnerability of pelagic, forage fish to visual predators (Hestir et al., 2016; Rypel et al., 2007) and by causing them to avoid sampling gear (Latour, 2016). We tested this hypothesis via multivariate autoregressive state‐space (MARSS) models that allowed testing support for different spatial structures of Longfin Smelt populations throughout the Estuary. Additionally, we hypothesized that (2) freshwater outflow would have strong positive effects on Longfin Smelt abundance. We tested this hypothesis via MARSS models with outflow as a covariate. Additionally, we hypothesized that (3) freshwater outflow would have a consistent and positive effect on Longfin Smelt populations across all time‐steps and habitats, demonstrating the importance of flow in determining suitable habitat (Grimaldo et al., 2020; Lewis et al., 2020). To test this hypothesis, we used DLMs that describe whether flow–Longfin Smelt relationships have changed in magnitude or sign over time. Finally, (4) we hypothesized that DLMs would reveal windows of flow sensitivity that time‐averaged techniques (such as MARSS) are unable to identify, thereby allowing us to more clearly understand when flow actions may be more effective in assisting with Longfin Smelt population recovery.

## METHODS

### Study area

The San Francisco Estuary is the largest drowned river valley estuary on the Pacific Coast of North America, extending from the Sacramento‐San Joaquin River Delta through the San Francisco Bay, and out to the Pacific Ocean. This highly engineered system provides water for approximately 25 million people and has many diversions to meet societal purposes for agricultural, industrial, and municipal uses (Cloern & Jassby, 2012). The Estuary is characterized by a Mediterranean climate, with distinct wet and dry seasons and considerable variation in the magnitude and timing of precipitation across years. Most precipitation in this system occurs in winter and spring, and these inputs, combined with tidal influences from the Pacific Ocean, are major sources of flow variability (Hutton et al., 2017; Kimmerer et al., 2013). Embayments span fresh, brackish, and meso/polyhaline conditions, with the extent and location of the salinity gradient shifting year to year with interannual variation in the magnitude and timing of river flows. The system is the focus of large‐scale conservation efforts, including environmental flow actions to support species of special concern (Cloern et al., 2010; CNRA, 2022; Feyrer et al., 2015).

### Longfin Smelt

The Longfin Smelt (*S. thaleichthys*) is an anadromous forage fish with a geographic range from California to Alaska, along the Pacific Ocean coast (Moyle, 2002). Longfin Smelt usually reach 80–150 mm FL, live for 2–3 years, and are semelparous. Larval and reproducing adults persist in estuaries (brackish to freshwater), while most adults move to the ocean by the end of age 1. This fish typically spawns between December and February, juveniles stay in low‐salinity habitats until spring, and then sub‐adults move into the bay and ocean during the summer. Although there is little research on the ocean stage of Longfin Smelt, recent studies have found that Longfin Smelt occur in shallow coastal waters in proximity to estuaries (Garwood, 2017; Young et al., 2024). Adults then return to the San Francisco Estuary in the fall for reproduction (Moyle, 2002). Movement from the ocean to spawning areas typically occurs between November and January, while reproduction typically occurs between December and April. Given substantial declines of Longfin Smelt populations at their southern range edge (Hobbs et al., 2017; Sommer et al., 2007), the San Francisco Bay‐Delta distinct population segment (DPS) of the Longfin Smelt was recently listed as Endangered under the United States Endangered Species Act (U.S. Fish and Wildlife, 2024).

### Biomonitoring data

**FIGURE 1:**
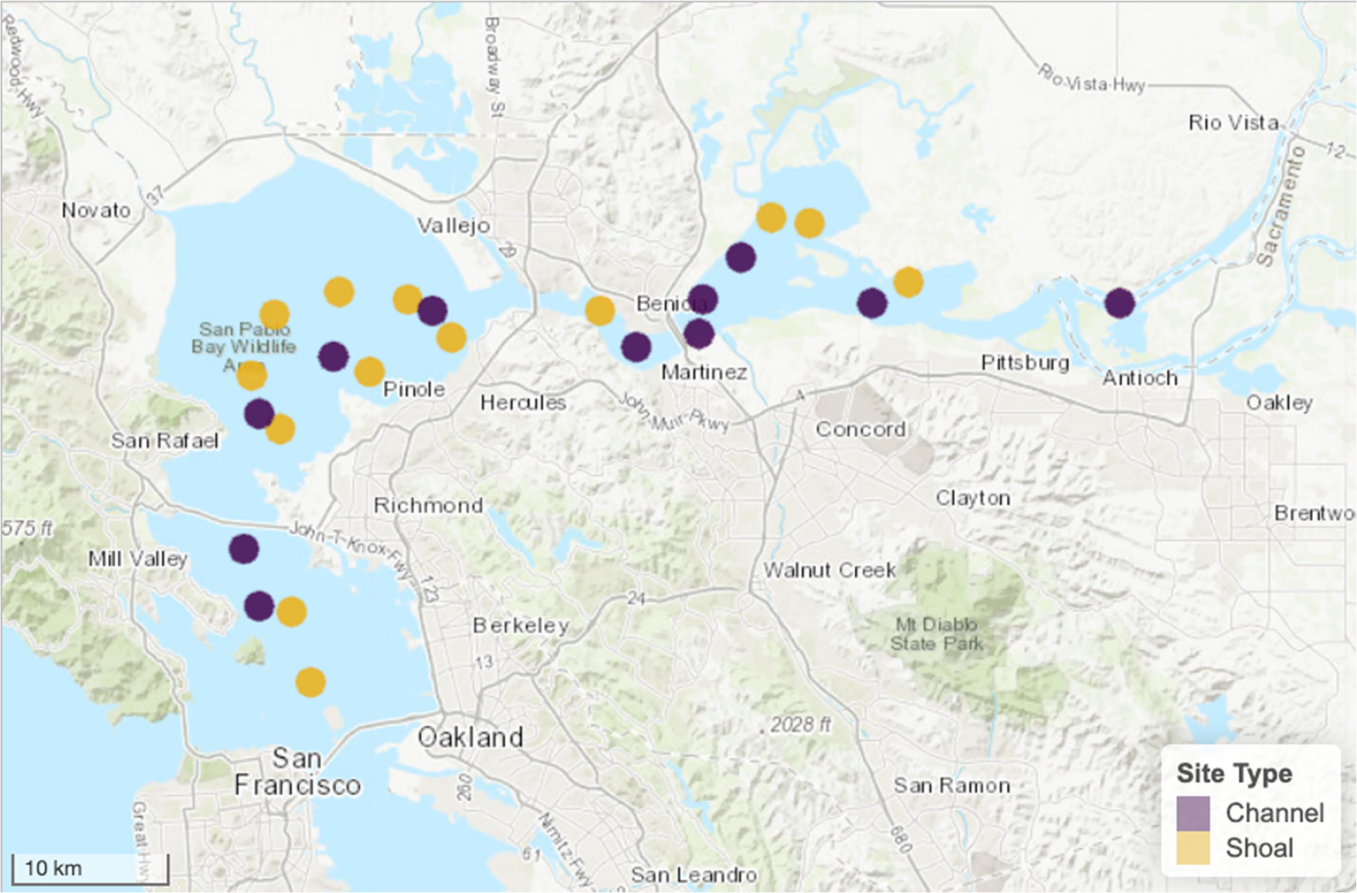
Map of the sampling locations of the Bay Study that passed the filtering protocol and were included in the modeling exercise. Colors represent stations located in channel versus shoal habitats.

Fish survey data used for this study were obtained from the California Department of Fish and Wildlife San Francisco Bay Study (hereafter, “Bay Study”), a long‐term monitoring program that samples fishes at fixed stations along the longitudinal salinity gradient of the Estuary. This monitoring program started in 1980 and takes place monthly at 35 stations along the South Bay, Central Bay, San Pablo Bay, Suisun Bay, and the Confluence of the Sacramento and San Joaquin rivers. Sampling takes place in channel and shoal habitats (Figure 1), and two trawling methods are used to capture fish: a midwater trawl that targets the pelagic environment, and an otter trawl that targets individuals near the bottom of the water column (i.e., the benthic environment). We used data from both trawl types in this study because Longfin Smelt can be found throughout the water column and have been known to migrate vertically, depending on environmental conditions (Bennett et al., 2002). At capture, all fish were counted, identified to species, and 50 randomly selected individuals were measured (fork‐length). When more than 50 Longfin Smelt were captured, the length distribution of the 50 sampled fish was used to estimate lengths of unmeasured fish. Fish age was assigned by length‐at‐month distributions (Baxter, 1999). These data were used to calculate catch per unit effort (CPUE) for annual age classes using an estimate of the volume of water sampled (in cubic meters) or area swept (in square meters) for the midwater and otter trawls, respectively. In this study, we analyzed age‐0 and age‐1+ Longfin Smelt catch data collected over four decades (1980–2020).

Additionally, we considered river flows entering the Estuary as estimated by the DAYFLOW model (https://data.cnra.ca.gov/dataset/dayflow). Specifically, we selected Net Delta Outflow, representing daily outflow estimated via a one‐dimensional hydrodynamic model, aggregated to a daily mean to capture the net difference between ebbing and flooding tidal flows. We later aggregated this variable at monthly and annual timescales to match the time step of the fish abundance data (see next section). We also assessed coherence between this Estuary‐wide modeled covariate and local, empirical measurements collected monthly as part of the Bay Study via Pearson correlation tests (Appendix S1: Figure S1).

### Data filtering and preparation

We developed a site‐filtering protocol to balance data density with spatial coverage to fulfill the data completeness requirements of the time‐series models for covariates. Specifically, we first examined the number of years of data available per station and the spatial clustering of high‐quality stations. We then filtered stations that had at least 50% completeness (following Colombano et al., 2022), applied a natural log transformation to CPUE values, and replaced monthly outliers (i.e., values ≥2 SDs from the mean) with linearly interpolated data from adjacent months. This process affected a small number of values (<5% of the total, both for otter and midwater trawls) and was implemented so that they would not disproportionally affect our models (Gitzen et al., 2012). Monthly CPUE for each of the 25 high‐quality stations and age class was aggregated at the annual scale to enter subsequent MARSS and DLM analyses. For age‐0 individuals (i.e., young‐of‐the‐year), annual averages were informed by the months of May–October; for age‐1+ fish, annual averages were calculated with a window spanning February–May. These windows concentrate most fish in each size class and are used by the Bay Study to develop annual abundance indices (Baxter, 1999). For the covariate (outflow), annual averages were calculated based on the months of January–June, the window when historically most of the annual Delta outflow takes place (77% on average).

### Data analysis

We first sought to visually explore temporal variation in Longfin Smelt abundance and raw associations between outflow and Longfin Smelt abundance. To this end, we plotted the Fall Midwater Trawl (FMWT) abundance index for Longfin Smelt against outflow, and we fitted separate regression lines for three time periods that represent different ecosystem states: before the system was invaded by non‐native clams (i.e., before 1987); after the clam invasions but before the POD took place (1988–2002); and during the most recent post‐POD period (2003–2020). For consistency with our Bay Study time‐series models, we restricted the visualization to start in 1980. The FMWT index aggregates mean catch per tow within regional areas, applies area‐specific weighting factors, and sums September–December indices to an annual index.

To address objective 1, on the spatial structure of age‐0 and age‐1+ Longfin Smelt populations in the Estuary, we first quantified the diversity of population trajectories over space (via Theil–Sen trend tests and coefficients of variation), and we analyzed the distributions of these variables, which captured population long‐term trends and variability, respectively. We then specified a set of grouping variables (region, lateral habitat, and vertical habitat) that could be potentially used to summarize population trends. These groupings then informed MARSS models representing different hypotheses about spatial structure such as panmictic versus region‐specific dynamics (Colombano et al., 2022; Pak et al., 2023; Ward et al., 2010). The region grouping specified whether stations were in one of three geographic categories: (1) Central Bay (seaward), (2) San Pablo Bay (middle), or (3) Suisun Bay and Confluence (landward). In turn, lateral habitat groupings included channel versus shoal, as identified by the CDFW Bay Study. Finally, vertical habitat grouped time series were collected via the otter trawl (in benthic environments) versus the midwater trawl (in pelagic environments). MARSS models are state‐space versions of MAR models that explicitly separate observation error from process error. Observation error represents uncertainty around measured values (e.g., arising from imperfect detection). In contrast, process error captures real variability in the population, such as fluctuations in abundance (and hence CPUE) arising from changes in recruitment or mortality. Estimating process error is valuable in conservation contexts because species with high process error (large intrinsic fluctuations in abundance) face greater extinction risk, especially when populations are small (Fournier et al., 2025; Holmes et al., 2012; Knape & de Valpine, 2012). In the matrix form, MARSS can be represented as:

(1) $$x_{t}=x_{t-1}+{\mathrm{Cc}}_{t}+w_{t},\text{where}\;w_{t}\sim \mathrm{MVN}\left(0,Q\right)$$

(2) $$y_{t}={\mathrm{Zx}}_{t}+v_{t},\text{where}\;v_{t}\sim \mathrm{MVN}\left(0,R\right)$$

where Equation (1) is the process model and Equation (2) is the observation model. Process error (**w**_**t**_), or unexplained variance, was estimated based on a multivariate normal distribution of mean 0 and covariance matrix **Q**. We specified **Q** as “diagonal and unequal,” thus assuming no covariance but allowing for the different states (e.g., locations within the estuary) to have different process error variances (i.e., diagonal values). In the observation model, **v**_**t**_ is a vector of observation error, estimated from a multivariate normal distribution of mean 0 and covariance matrix **R**. In our case, we assumed no covariance in observation error, and observation error variances (i.e., the diagonal values in the **R** matrix) were estimated for each region and gear type. Our observations (annual Longfin Smelt CPUE) were station‐, gear‐, and age‐specific, and the **Z** matrix connected these observations (**y**_**t**_) to the “hidden” or observation error‐free states (**x**_**t**_). In turn, covariate data (annual Delta outflows) entered the state model as **c**_**t**_. We fixed **u** (population growth rate) and **a** (scaling parameter) to zero to allow for direct comparison with the DLM, where these parameters are conventionally set to zero (Holmes et al., 2012).

A total of six model structures were fitted to each Longfin Smelt dataset (age‐0 and age‐1+), each representing a spatial hypothesis via the **Z** matrix: estuary‐wide trends (i.e., a single state); vertical habitat‐specific trends (otter trawl vs. midwater trawl; i.e., two states representing benthic vs. pelagic environments, respectively); region‐specific trends (i.e., three states); vertical habitat and region‐specific trends (i.e., six states); lateral habitat‐specific trends (channel vs. shoal; i.e., two states); and lateral and vertical habitat‐specific trends (i.e., four states) (see details in Table 1). This first set of models included no covariate effects or covariate data (i.e., **Cc**_**t**_ in Equation 1 was set to zero). Model support was compared via difference in corrected Akaike information criterion between models (AIC_c_), and the states of the MARSS best model (i.e., the states representing true population fluctuations after accounting for observation error) were extracted for posterior analysis to describe the effects of outflow (via DLM).

**TABLE 1:** Akaike information criterion (AIC) table for age‐1 and 1+ fish, listing hypothesis tested, AIC, and difference in corrected AIC (ΔAIC_c_) of models run in the first multivariate autoregressive state‐space (MARSS) model testing for the number of states that best describe the population structure.

Hypothesis	Description	No. states	Age‐0 AIC_c_	Age‐0 ΔAIC_c_	Age‐1+ AIC_c_	Age‐1+ ΔAIC_c_
H1: Estuary‐wide	One state across entire estuary	1	2562.49	1759.19	2210.86	1962.01
H2: Vertical habitat	Gear type‐specific states (otter trawl or midwater trawl)	2	894.03	90.74	453.48	204.64
H3: Region	Region‐specific states: (1) Central Bay; (2) San Pablo Bay; (3) Suisun Bay and Confluence	3	2562.32	1759.03	2213.22	1964.37
H4: Vertical habitat‐Region	Combination of gear and region‐specific states (three regions and two gear types)	6	927.26	123.97	482.29	233.45
H5: Lateral habitat	Lateral habitat‐specific states (channel or shoal)	2	2560.81	1757.52	2051.35	1802.59
**H6: Lateral and vertical habitat**	**Combination of lateral and vertical habitat states (channel or shoal; midwater trawl or otter trawl)**	**4**	**803.29**	**0**	**248.84**	**0**

*Note*: The bolded hypothesis is the one with the lowest value AIC_c_ (at the bottom of the table).

To address objective 2, we then modeled Longfin Smelt time series via MARSS models, estimating time‐averaged outflow effects using the **C** matrix formulation. To do this, we used the best model describing trends in age‐0 and age‐1+ from objective 1, and included a flow covariate (**c**_**t**_ in Equation 1) to determine the general relationship between Longfin Smelt and outflow (**C** in Equation 1, representing “time‐averaged” flow effects).

To address objective 3, on the potential time‐varying effects of flow on Longfin Smelt dynamics, we used DLMs, which are structurally similar to MARSS but with a time‐varying **Z** matrix. Specifically, the relationship between flow and Longfin Smelt was modeled via a time‐varying or “evolution” equation, estimating a random walk for each parameter (slope and intercept). Like MARSS, DLMs account for temporal autocorrelation patterns inherent to ecological time‐series data and can detect changes in ecological relationships while simultaneously considering population‐level constraints (i.e., the effects of the population on itself) and external environmental forcings (i.e., the effects of the environment on the population). The observation‐error‐free CPUE states from MARSS models were used as inputs to DLMs with a multivariate structure:

(3) $$x_{t}=x_{t-1}+w_{t},\text{where}\;w_{t}\sim \mathrm{MVN}\left(0,Q\right)$$

(4) $$y_{t}=Z_{t}x_{t}+v_{t},\text{where}\;v_{t}\sim \mathrm{MVN}\left(0,R\right)$$

where outflow data at year *t* were added to the model in Equation (4) via **Z**_**t**_, an array with the matching covariate data at each time step. In our case, **Z**_**t**_ was a 4 × 8 × 41 array, as we calculated covariate effects for each of the four habitat groups or states, entering DLM as observations (i.e., channel‐midwater, shoal‐midwater, channel‐otter, shoal‐otter). For each habitat group, DLMs then estimated two time‐varying regression parameters in **x**_**t**_, one for the slope and the other for the intercept (Equation 3). Process error (**w**_**t**_) was drawn from a multivariate normal distribution with a mean of 0 and a covariance matrix of **Q**. Similarly, observation error was drawn from a multivariate normal distribution with a mean of 0 and a covariance matrix of **R**, which was specified as “diagonal and unequal” (i.e., allowing for independent observation error variances across groups). Three sets of DLMs were run with this structure, with two different response variables and two different predictors: (1) age‐0 CPUE with contemporary outflow (January–June annual averages), (2) age‐1+ CPUE with contemporary outflow, and (3) age‐1+ CPUE with lagged outflow (1‐year lag, capturing previous‐year flow effects on current‐year population size). The rationale for these different models was to understand whether flow could affect Longfin Smelt recruitment (age‐0), growth and survival (age‐1+), or abundance via recruitment effects from the previous year (age‐1+ lagged flow). The Broyden–Fletcher–Goldfarb–Shanno (BFGS) algorithm was used as an optimization algorithm to ensure convergence (Auvinen et al., 2009).

Finally, objective 4 allowed comparing inferences from MARSS and DLM outputs, and determining whether allowing flow effects to be time‐varying would change our understanding of Longfin Smelt population dynamics. To this end, we plotted the “time‐averaged” flow effects provided by MARSS to those obtained via DLMs, which had a flow effect for each time step. To allow for direct comparison between the two methods, we examined time‐varying DLM slopes in different ways: by averaging them over time, and by identifying its maximum and minimum slope (and associated 95% CIs), representing points in time when flow–fish relationships were strongest (or weakest) in either direction. This exercise allowed us to further understand whether, at some points, the maximum and minimum values from the DLMs were close to the estimates provided by MARSS.

## RESULTS

### Longfin Smelt abundance index, population structure, and trends (objective 1)

**FIGURE 2:**
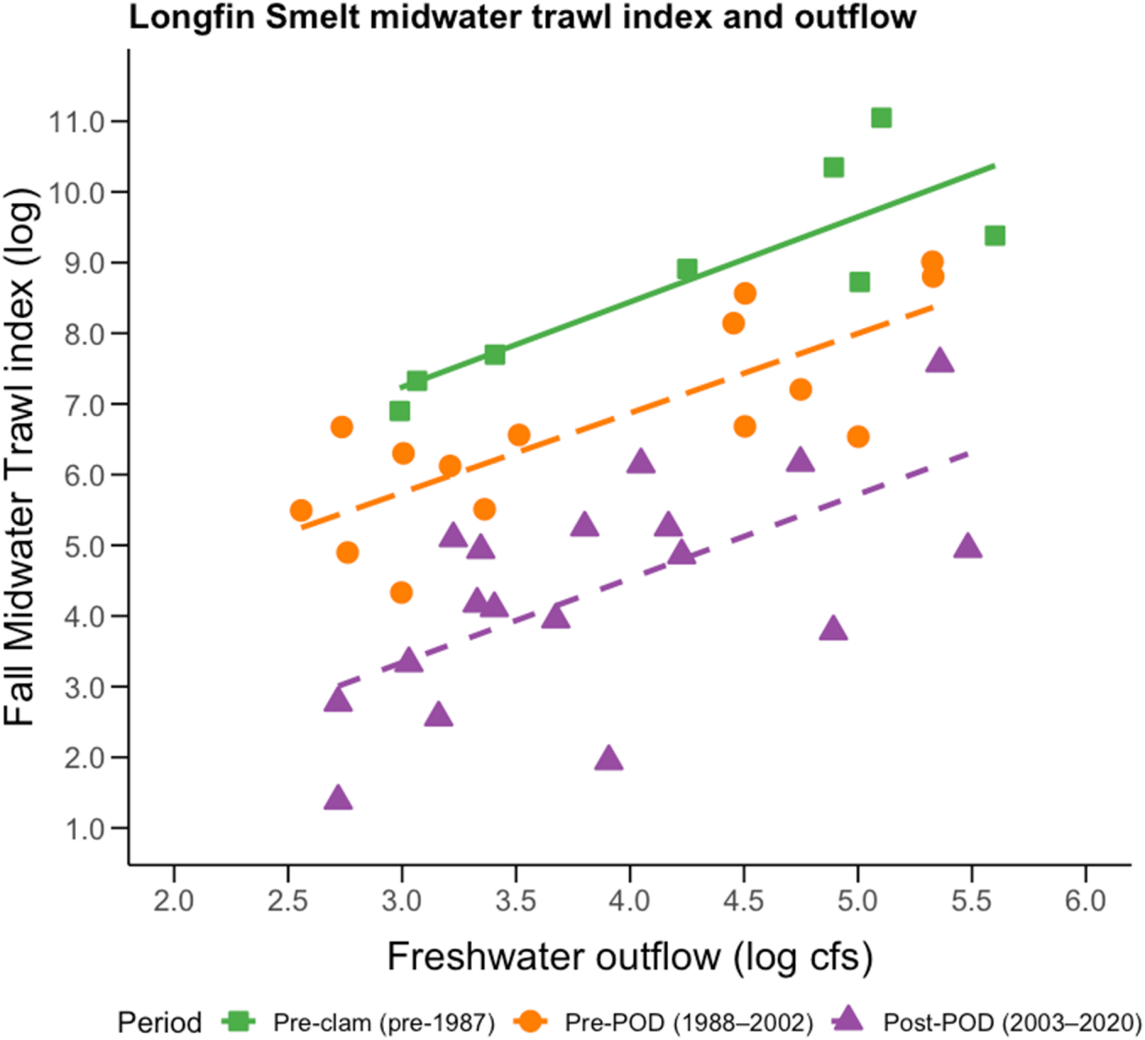
Associations between freshwater outflow (cubic feet per second; January–June mean) and the Fall Midwater Trawl (FMWT) abundance index of Longfin Smelt in three pre‐specified time periods that separate major ecosystem state shifts in the San Francisco Estuary: Before the invasions of the non‐native clams (i.e., pre‐1987), after the invasions but before the Pelagic Organism Decline (POD; 1988–2002), and in the most recent post‐POD period (2003–2020). Lines are simple linear regressions (pre‐clam: slope = 1.205, *R* ^2^ = 0.71, *p* = 0.0087; pre‐POD: slope = 1.125, *R* ^2^ = 0.637, *p* = 0.0003; post‐POD: slope = 1.19, *R* ^2^ = 0.404, *p* = 0.0046).

Annual CPUE of Longfin Smelt (all ages) showed strong interannual swings (CV = 105.73%), with interannual variation in abundance (aggregated at the whole‐estuary scale) being moderately associated with interannual variation in river outflows. Raw flow–ecology associations using the Longfin Smelt abundance index were consistently positive, but suggested that Longfin Smelt abundance tended to decrease across the prespecified time periods, and that the explanatory power of flow weakened over time, with coefficients of determination (*R* ^2^) being highest in the early period (pre‐clam invasion) and weakest in the most recent post‐POD period (Figure 2). This finding confirmed the need to use time‐series models (MARSS and DLMs) to further examine time‐averaged and time‐varying effects of flow (objective 2 as mentioned subsequently here).

**FIGURE 3:**
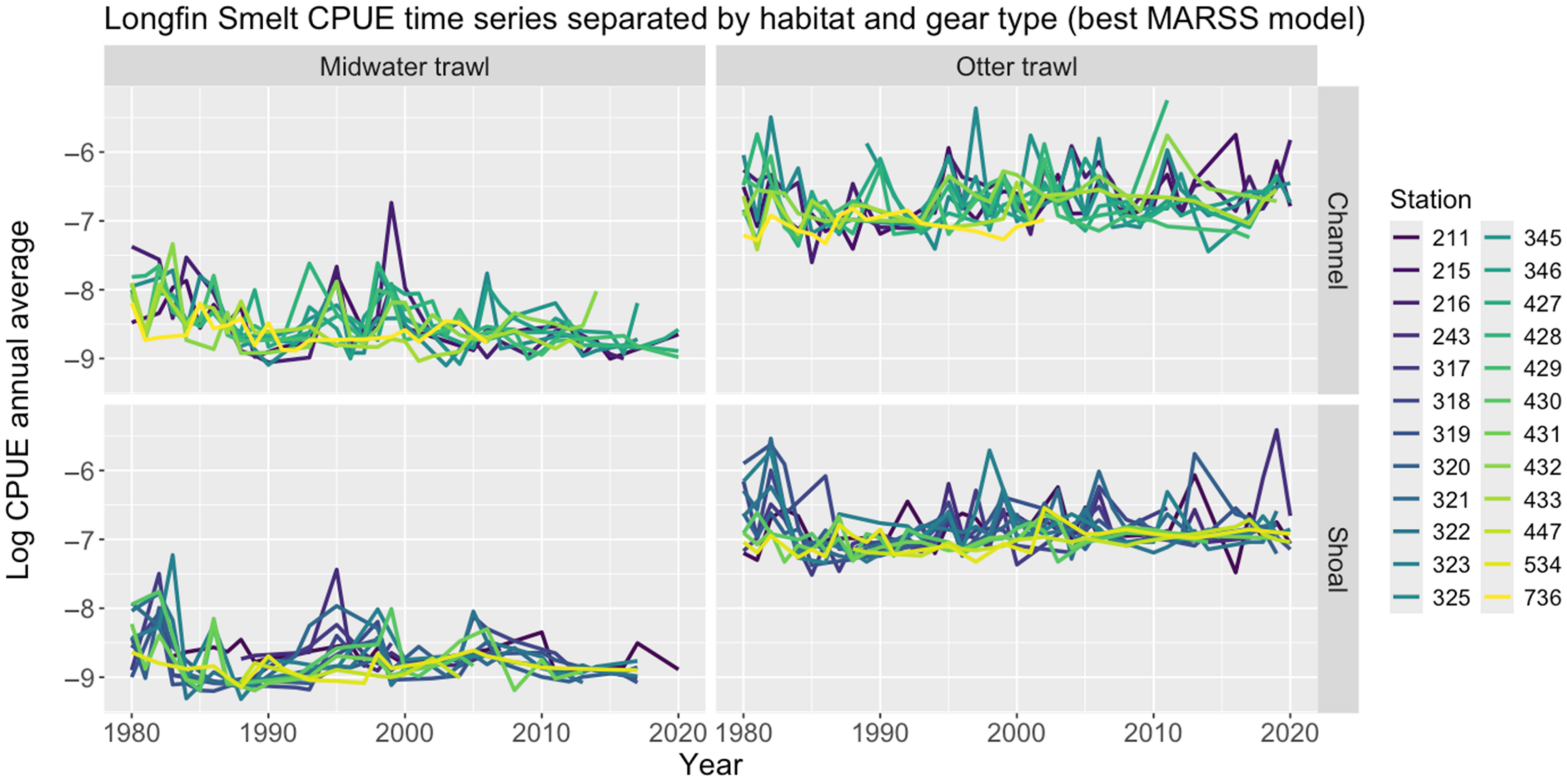
Annual average catch per unit effort (CPUE) of Longfin Smelt, displayed by vertical habitat (midwater trawl, capturing the pelagic environment vs. otter trawl, capturing the benthic environment) and lateral habitat (channels vs. shoals), representing outputs of the multivariate autoregressive state‐space (MARSS) model that was best supported in our model comparison exercise. Missing years were filled via linear interpolation for visualization purposes only. Values were natural‐log transformed, and each line represents an individual station (see Figure 1).

We then examined population structure and trends across the Estuary via MARSS models. We found that models with different numbers of states, each representing a hypothesis about spatial population structure, showed that a combination of lateral and vertical dimensions of habitat structure (i.e., channel vs. shoal, midwater trawl vs. otter trawl) best described the diversity of Longfin Smelt population trajectories (Table 1, hypothesis 6). Notably, this hypothesis received the strongest support for both the young‐of‐the‐year (age‐0) and the older cohort (age‐1+). The data did not support further simplification of population trajectories (e.g., a single estuary‐level state; hypothesis 1) or more complex structures (e.g., six vertical habitat‐region‐specific states; hypothesis 4; Table 1). When examining both CPUE and the population states (i.e., the MARSS outputs free of observation error), we observed that in both channel and shoal habitats, CPUE was higher in benthic habitats (otter trawl) than it was in pelagic habitats (midwater trawl; Figure 3). Overall, these results were in line with our prediction for objective 1 on the importance of vertical habitat as a variable explaining diversity in population trends.

**FIGURE 4:**
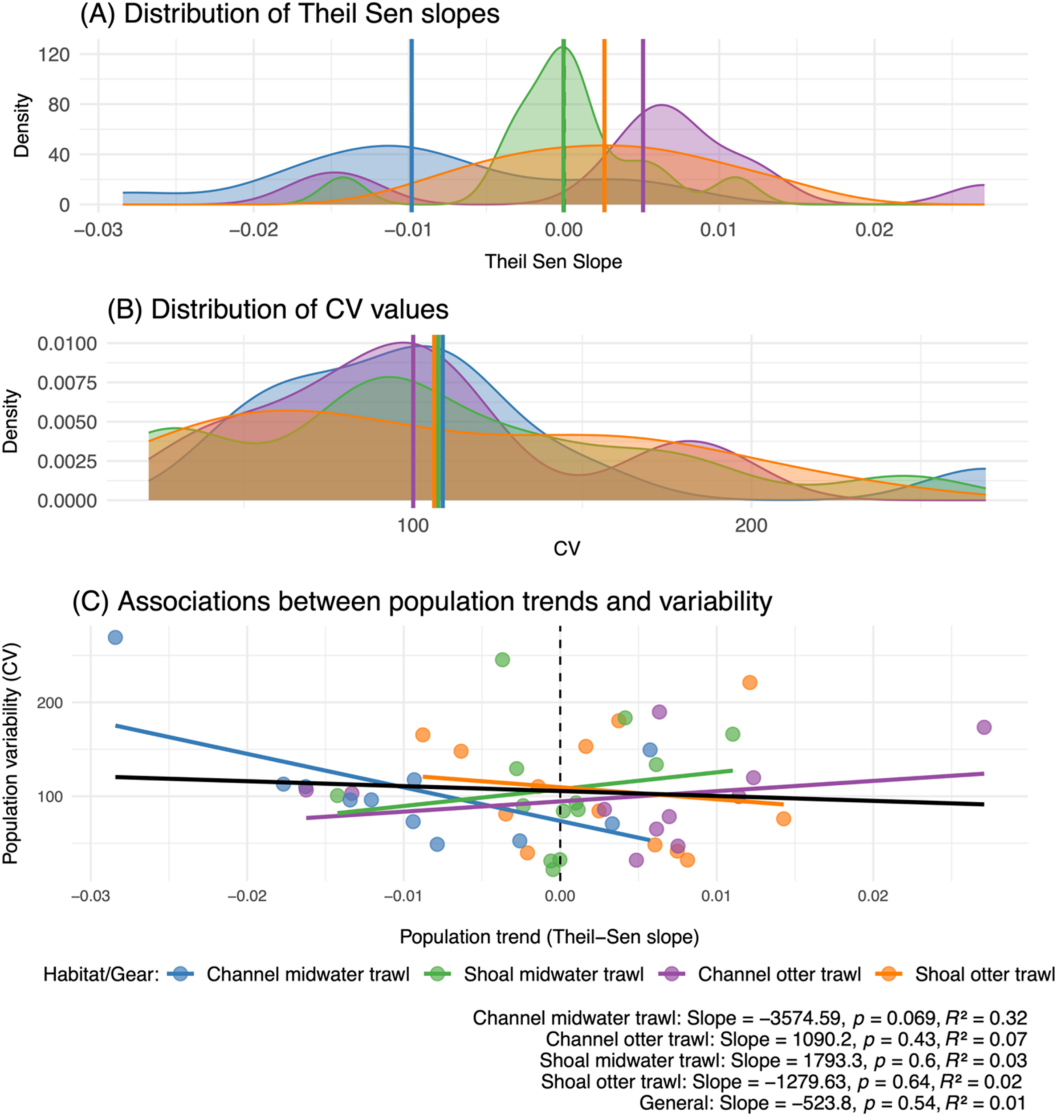
Distribution of population trends (A, Theil–Sen slopes), variability (B, CV), and the association between the two variables (C) across the studied stations, plotted by lateral and vertical habitat groups (channel, shoal, midwater trawl, otter trawl). In panels (A) and (B), vertical lines show group‐level means. In panel (C), we show group‐specific slopes (in colors) versus a general slope pooled across groups (in black), and the equation of each simple linear regression.

When we examined the diversity of long‐term population trajectories across sampling stations via a robust trend estimator (Theil–Sen trend), we confirmed that, despite a wide range of values, the lateral and vertical dimensions of habitat structure (i.e., shoal vs. channel, and pelagic vs. benthic habitats) tended to be associated with different population trajectories, with trends declining markedly in channel‐midwater (pelagic) habitats (Figure 4A). While CV ranges were similar across habitat groups (Figure 4B), within‐group CV was associated with long‐term population trends in channel‐midwater (pelagic) habitats, revealing that stations exhibiting the steepest declining trends also tended to have higher temporal variability in that habitat type (Figure 4C). Overall, we observed negative trends in 48% of the stations, and negative trends were 1.6‐fold more likely to be statistically significant than positive trends were (i.e., 39% vs. 24% respectively) (Appendix S1: Table S1).

### Flow effects on Longfin Smelt population dynamics (objective 2)

The second set of MARSS models, which included river outflows as a covariate, revealed that age‐0 Longfin Smelt in channel‐midwater (pelagic) habitats responded positively to freshening events (i.e., high‐outflow years). The other three states showed inconsistent responses to flow, with CIs for the covariate (flow) effect including zero (Appendix S1: Figure S2). In turn, age‐1+ Longfin Smelt captured by midwater trawls, both in channel and in shoal habitats, responded positively to same‐year outflow, but this relationship was not observed in benthic habitats (based on otter trawl data). When assessing potential lagged effects of flow on age‐1+, we found significant flow effects for channel populations in midwater (pelagic) habitats, likely reflecting flow effects on older fish via recruitment from the previous year.

### Time‐varying effects of flow (objective 3)

**FIGURE 5:**
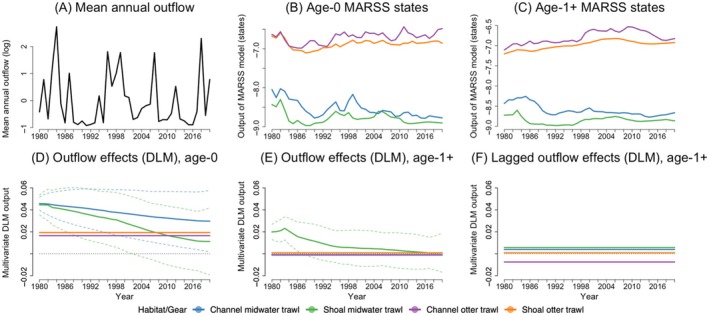
Temporal trends in river outflow (A), Longfin Smelt states from the multivariate autoregressive state‐space (MARSS) model (B, C), and outflow effects (D–F) based on dynamic linear models (DLMs) for age‐0 Longfin Smelt, and for age‐1+ using same‐year flow and previous‐year flow; 95% CIs representing uncertainty in flow effects are shown via dashed lines.

The time‐varying DLM slopes represented the strength of the relationship between outflow (Figure 5A) and the Longfin Smelt CPUE states for each age class (Figure 5B,C). Three of the 12 calculated DLM slopes exhibited time‐varying characteristics, while the rest of the relationships were best described as static and often weak (Figure 5D–F). Age‐0 fish in channel‐midwater and shoal‐midwater habitats had similarly positive initial slopes, but the latter decreased in slope more rapidly (Figure 5D). Moreover, the lower CI for fish in shoal‐midwater habitats crossed the *y* = 0 line around 2002, denoting uncertain flow effects after that year. However, the slightly positive maximum‐likelihood slope for both groups suggests that as outflow increases, Longfin Smelt populations also tended to increase. Positive, yet weak, flow effects were also observed with otter trawl data, reflecting fish dynamics in the benthic environment, both in channel and in shoal habitats, with consistent values around 0.02 throughout the four decades (Figure 5D).

When examining responses of age‐1+ fish, we found that contemporary flow affected CPUE in shoal‐midwater habitats in a time‐varying way, starting positively but becoming uncertain by 1988 (Figure 5E). Age 1+ fish in all other habitat groups showed near‐zero, static responses to outflow. In turn, previous‐year outflow (i.e., covariate values lagged by one year) never showed time‐varying effects on fish counts, and slopes were either slightly positive (e.g., in shoal‐midwater [pelagic habitats]) or slightly negative (e.g., in channel‐otter (benthic) habitats; Figure 5F).

Overall, our results did not match predictions for objective 3, since flow effects on Longfin Smelt populations often started strongly but then decreased, or remained constant around a low value, over the four decades of study.

### Does a time‐varying approach change our understanding of Longfin Smelt population dynamics and its drivers? (objective 4)

**FIGURE 6:**
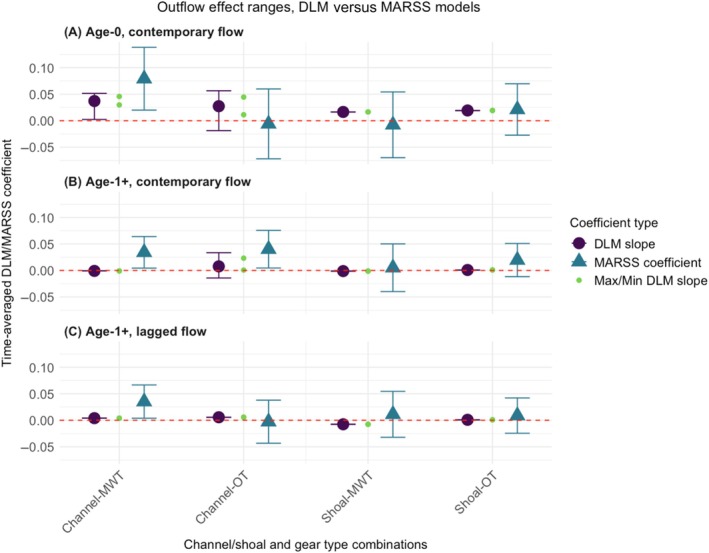
Comparison of flow effects estimated by the two competing modeling approaches: multivariate autoregressive state‐space (MARSS) model with covariate versus dynamic linear model (DLM) for channel, shoal, otter trawl (OT), and midwater trawl (MWT). For DLM, CIs were obtained from the maximum and minimum slopes through the entire time series. The upper 95% CI for the maximum slope and the lower 95% CI for the minimum slope bound the time‐averaged DLM slope (shown as teal triangles). The most extreme maximum and minimum slopes were also plotted (as smaller green circles) to show the whole range of variation in estimated slopes, and determine whether at some point these estimates fell close to the MARSS “time‐averaged” estimate. The red dashed line at *y* = 0 represents no flow effects. The three panels represent outputs from different pairs of MARSS‐DLMs (fitted with different data), namely: (A) Age‐0 Longfin Smelt and contemporary flow; (B) Age‐1+ Longfin Smelt and contemporary flow; and (C) Age‐1+ Longfin Smelt and previous‐year flow (i.e., lagged by one year).

When comparing DLM flow effects averaged across the time series to flow effects as estimated by MARSS (which assumes static relationships), we found important differences (Figure 6). Most outputs from both models overlapped in CIs, and the most extreme flow effects estimated by the DLM (i.e., the maximum and minimum value of the slope for each group) tended to fall within the 95% MARSS CIs (Figure 6). However, only about 30% of the outputs returned the same sign, suggesting that these two approaches may often lead to inferences that differ qualitatively from each other.

For age‐0 Longfin Smelt in channel‐pelagic (midwater) habitats, the lower CI of the MARSS coefficient did not capture as much variability as the averaged DLM slope (Figure 6A), and a similar pattern was apparent for age‐1+ Longfin Smelt with contemporary flow, where the CI for DLMs included negative values (suggesting negative responses to flow) while MARSS did not (Figure 6B). In turn, both methods provided the same sign on flow effects when examining age‐0 fish in channel‐midwater (pelagic) and shoal‐otter (benthic) habitats; age‐1+ fish in channel‐otter (benthic) habitats, and age‐1+ fish responding to lagged outflow (i.e., flow conditions in the previous year; Figure 6A–C). Notably, in many cases DLMs were unable to establish a time‐varying relationship (or CIs associated with flow effects), but MARSS did estimate flow effects (Figure 6). This divergence likely suggests limitations arising from statistical power. Overall, our results match our predictions for objective 4, since some DLMs provided time‐varying flow effects that differed in magnitude and even sign from those estimated via the more traditional, time‐averaged (MARSS) approach.

## DISCUSSION

Freshwater flow is a key driver of estuarine dynamics, shaping salinity gradients, nutrient availability, and habitat characteristics (Sofi et al., 2020). In the San Francisco Estuary, interannual variability in freshwater flow controls recruitment of many invertebrate taxa and fish, including the federally endangered Longfin Smelt (*S. thaleichthys*) (Kimmerer, 2002a; Stevens & Miller, 1983; Tamburello et al., 2019). Here, we challenged the “stationarity assumption” of flow–ecology relationships and found that the strength of flow effects is weakening for Longfin Smelt in the San Francisco Estuary. Non‐stationarity—when environmental relationships shift over time due to factors like invasive species, habitat degradation, or climate change—can obscure key flow–abundance patterns, reducing the effectiveness of static models as a predictive tool (Poff, 2018; Tonkin et al., 2019). For example, although our ad hoc simple linear regressions showed positive flow–abundance association across the three prespecified time periods (Figure 2), our time‐series approaches yielded new insights—namely, how the strength of the relationship changed through time, across habitats, and life stages.

In our case, the 1987 invasion of the overbite clam (*Potamocorbula amurensis*) profoundly altered the pelagic food web (Winder & Jassby, 2011), and broader ecosystem disruptions associated with the POD in the early 2000s have further impacted productivity (Sommer et al., 2007), stimulating this investigation into non‐stationary flow–abundance relationships. Using time‐varying DLMs and “time‐averaged” MARSS models on the same biomonitoring data, we investigated how Longfin Smelt has been responding to estuarine outflow over the last four decades (1980–2020). Our analyses revealed that population trends differed across lateral and vertical dimensions of habitat, captured here by sampling pelagic and benthic environments (via midwater vs. otter trawls) in channels and shoals. While positive outflow effects were evident from both approaches, DLMs revealed that the strength of these effects has been decreasing over time in some cases—particularly for age‐0 Longfin Smelt collected at pelagic‐shoal and pelagic‐channel sites. Meanwhile, older fish (age‐1+) only responded to outflow in a time‐varying way in pelagic‐shoal sites. Finally, when comparing the DLM versus MARSS approaches, we found that agreement was found in only 30% of our models, underscoring the importance of accounting for non‐stationarity in ecological relationships. Our work highlights the need for time‐varying approaches to better understand evolving flow–abundance dynamics of endangered species, and guide more effective adaptive management for them.

### Weakening responses of Longfin Smelt to river outflows

Our DLMs revealed weakening—though still positive—outflow effects on Longfin Smelt abundance in certain habitats. Flow–ecology relationships were strongest in pelagic‐channel habitats, and generally weaker in shoal habitats. This pattern could be due to ongoing decline in turbidity in the Estuary (Lee et al., 2021; Schoellhamer, 2011), as this weakening effect was most pronounced in pelagic habitat where turbid conditions historically provided refuge from predation (Barnard et al., 2013). Alternatively, Longfin Smelt may avoid habitats with low turbidity (high water clarity), resulting in lower abundance in these habitats (Latour, 2016). Nevertheless, the relationship between outflow and age‐0 Longfin Smelt continues to be positive, consistent with prior studies (Hartman et al., 2024; Kimmerer & Gross, 2022; Nobriga & Rosenfield, 2016; Tamburello et al., 2019). When examining older fish (age‐1+), we also observed weakening responses to outflow in pelagic‐shoal habitats. One possible explanation is the reduction in high‐flow pulses in the San Francisco Estuary, which may have historically cued ocean‐dwelling adults to return to the Estuary (Hutton et al., 2017; Nobriga & Rosenfield, 2016).

We did not find evidence of time‐varying relationships between age‐1+ Longfin Smelt and lagged outflow, implying that flow–ecology relationships apparent at the age‐0 life stage are diminished later in life (in agreement with Nobriga & Rosenfield, 2016). This pattern may indicate that other drivers, such as habitat degradation, predation, or ocean mortality, are important for Longfin Smelt survival from age‐0 to later life stages (MacNally et al., 2010; Thomson et al., 2010), as has been found in other anadromous fishes (Adeva‐Bustos et al., 2019; Kennedy et al., 2008). Similarly, life stage‐specific drivers have been documented in anadromous salmonids and Delta Smelt populations, which are also heavily reliant on estuarine habitats during part of their life cycle (Finger et al., 2017; Fisch et al., 2011; Kareiva et al., 2000). These propagating effects may be difficult to quantify in small‐declining populations and may be contributing to weakening relationships (Belovsky et al., 1994).

To further clarify these dynamics, we analyzed year‐to‐year variability in Longfin Smelt abundance (Appendix S1: Figure S3). This supplementary analysis showed a marginal increase in annual variability over time (Appendix S1: Figure S3a), which did not appear to be related to mean abundance (Appendix S1: Figure S3b). Thus, we contend that weakening flow–abundance relationships may reflect genuine ecological shifts rather than statistical artifacts. Additionally, because our modeling approaches (MARSS and DLM) account for process errors, we were able to distinguish the effects of the population on itself from the exogenous effects of flow on the population.

Importantly, we did not find evidence for distinct step changes in the relationship between flow and Longfin Smelt abundance, as could be expected from recognized state shifts in the Estuary (e.g., after the clam invasion; Sommer et al., 2007). Rather, we observed a gradual decrease in the positive effect of flow on Longfin Smelt abundance. One plausible explanation for this pattern is the long‐term degradation of habitat quality and availability. The gradual loss or alteration of habitats that historically supported Longfin Smelt may mean that freshwater outflow no longer provides the same ecological benefits it once did, such as access to low‐salinity refuge, turbid foraging areas, or shallow nursery habitats, except during unusually high‐flow years when shoals and tributaries become inundated (Grimaldo et al., 2020; Hartman et al., 2024; Lewis et al., 2020). An alternative explanation is that Allee effects could be emerging as population sizes reach critically low levels. Although detecting Allee effects is inherently difficult because they act most strongly at low densities, they can amplify the vulnerability of small populations by reducing individual survival or reproductive success when abundance falls below a threshold (Knape & de Valpine, 2012). Supporting this possibility, Longfin Smelt in the San Francisco Estuary exhibit the lowest genetic differentiation across their range (Gilroy et al., 2012; Saglam et al., 2021), suggesting limited subpopulation structure and heightened susceptibility to demographic stochasticity.

### Implications for conservation

Our results also have important implications for monitoring, management, and restoration strategies to support Longfin Smelt recovery. MARSS models showed that both age‐0 and older Longfin Smelt exhibit distinct population trajectories across habitat types. These habitat‐specific trends suggest that lateral (channel vs. shoal) and vertical (pelagic vs. benthic) habitats should be treated as separate monitoring strata, rather than simply as grouping sites by regions (embayments), as is currently common practice. Accounting for these habitat dimensions would allow monitoring programs to better detect mechanistic responses to environmental drivers such as flow and water clarity, improve estimation of habitat‐specific productivity, and refine restoration targets. For management, this approach highlights the importance of conserving a mosaic of habitat types—particularly shallow shoals and tidal marsh channels—that support different life stages and functions within the Longfin Smelt life cycle. Integrating such habitat‐stratified design into monitoring could help ensure that outflow and habitat restoration efforts jointly enhance estuarine resilience and population recovery potential.

The steep declines in flow benefits revealed by DLM analyses for shoal habitats underscore the need to prioritize habitat restoration efforts in these habitats. For shoal habitats, management actions may focus on vegetation removal to restore shallow pelagic open‐water habitats and increase turbidity and prey availability, thus reducing predation risk (Boyer et al., 2023; Christman et al., 2023; Feyrer et al., 2021). Moreover, restoration of channel habitat via flow pulses, and associated increases in sediment delivery, phytoplankton and zooplankton production, could also benefit Longfin Smelt (Grimaldo et al., 2017). Because uncertainty around the flow–ecology relationship is increasing over time in all model outputs, flow or habitat interventions should be paired with expanded, high‐intensity monitoring across the habitat types utilized by Longfin Smelt and other native species. Such adaptive monitoring will be critical for evaluating the effectiveness of restoration actions and refining future management strategies. Previous studies have shown that freshwater flow is linked to benefits early in the Longfin Smelt lifecycle (Nobriga & Rosenfield, 2016), providing a foundation for targeted flow management actions in the Estuary. Past flow actions in the Estuary have shown that freshwater flow pulses can support increased low‐salinity rearing habitats and thus benefit endangered fish (Frantzich et al., 2021). Such flow pulses in the Estuary have been shown to increase primary production and zooplankton abundance, which are critical for Longfin Smelt diet (Barros et al., 2024; Sommer et al., 2020). Increasing the magnitude and/or frequency of flow pulses could bolster Longfin Smelt populations via multiple mechanisms by which flow improves habitat conditions, such as food subsidies, turbidity, or salinity (Sommer et al., 2020).

Finally, the weakening relationship between flow and Longfin Smelt suggests a pressing need to reassess freshwater outflow allocations for the species. While past studies have shown that managed flow pulses can produce positive ecological responses (Sommer et al., 2020), status quo outflows may not be sufficient to benefit Longfin Smelt—contributing to the declining flow–abundance. Strategically timed flow actions could improve degraded habitat conditions by increasing turbidity preferred by Longfin Smelt and other native species (Kimmerer & Gross, 2022) during key life stages. The steeper declines in midwater trawl catches may indicate that Longfin Smelt are seeking refuge in deeper, benthic habitats sampled by the otter trawl surveys. Thus, developing conservation actions that enhance habitat quality in deep‐water habitats, in tandem with flow management, may help preserve Longfin Smelt in the Estuary. Together, these findings emphasize that sustaining Longfin Smelt in a changing estuary will require an integrated approach—one that combines strategic flow management, habitat restoration across depth and lateral gradients, and adaptive, habitat‐stratified monitoring to track ecological responses and guide effective recovery actions.

### Embracing non‐stationarity in flow–ecology research

Strong flow–ecology relationships are underpinned by adaptations to the natural flow regime (Lytle & Poff, 2004) and have often become the mechanistic basis for flow management actions. Poff (2018) suggested that such relationships may not always be static, highlighting the need to evaluate this common assumption in flow management. Flow–ecology relationships have been shown to change when strong alterations in the environment are not captured in historical data (Poff & Zimmerman, 2010), when a new limiting factor appears relative to the historical period that prevents flow from continuing to be as effective as it was (MacNally et al., 2010), or when the food web undergoes drastic changes in structure or dynamics (Rogers et al., 2024).

Our work contributes to a small but growing literature exploring time‐varying relationships between the environment and biological endpoints. One such study examined phytoplankton dynamics along the French coast and identified the usefulness of DLMs in identifying significant phases within their time series (Soudant et al., 1997). This observation laid the groundwork for using time‐varying relationships to identify windows of vulnerability between physical and biological relationships (in particular, between wind and phytoplankton). Liu et al. (2021) used a related methodology (changing point analysis) as a tool to estimate the point at which the mean or variance of a time series of observations changed. Working with zooplankton communities, they showed that extreme floods were more impactful than storm surges to community resilience and associated recovery in Galveston Bay, Texas. Their conclusions highlight the usefulness of time‐varying approaches to identify the potential impact of disturbances operating at different timescales. In sum, other time‐varying approaches have found similar utility to our DLMs in answering questions about recruitment, flow–ecology relationships, and impacts of anthropogenic change.

### Limitations and future directions

Our study is one of the first to use DLMs to document flow–ecology relationships in an estuarine system, which revealed weakening flow–ecology relationships. However, certain limitations to our study deserve mention. The time‐step we used (annual) could have missed intra‐annual responses of Longfin Smelt to flow; thus, finer scale analyses could reveal flow–ecology relationships relevant at seasonal or monthly timescales that could aid in Longfin Smelt conservation (Colombano et al., 2020; Hobbs et al., 2019). Additionally, our dataset did not capture the entire distribution range of Longfin Smelt in the Estuary (Lewis et al., 2020; Young et al., 2024). Future research should consider newly restored areas in the Estuary (e.g., in the South Bay) that may act as population refuges and metapopulation sources. Finally, additional examination is warranted of proximate environmental variables that may be driven by, or correlated with, intra‐ and interannual variation in flow, such as salinity, turbidity, and food availability (Barros et al., 2024). Overall, long‐term monitoring programs enable the use of time‐series methods to understand fish population dynamics and associated drivers (Tempel et al., 2021). Despite the limitations inherent in any dataset, the quantitative approach we employed here holds promise and could be transferred to other systems where adequate, long‐term spatiotemporal data exist.

The San Francisco Estuary has shown strong non‐stationarity in hydrological and biological conditions over the last decades, like many other estuaries globally (Kousali et al., 2022; Wang et al., 2021). Notably, these trends will likely continue (Knowles & Cayan, 2002). Although flow–ecology approaches assuming static relationships may not be useful given the strong variability and changes impacting this ecosystem, flow continues to be a key variable for understanding and managing estuarine dynamics. By recognizing that flow conditions, ecological conditions, and flow–ecology relationships can themselves change, we contend that new quantitative tools may provide more precise inferences on the effects of changing river flow regimes. A better, more mechanistic understanding of the effects of river flow on estuarine habitats and populations could help improve management actions and conservation outcomes.

## CONFLICT OF INTEREST STATEMENT

The authors declare no conflicts of interest.

## Supporting information


Appendix S1.

## Data Availability

Data (Saffarinia et al., 2025a) are available in Dryad at https://doi.org/10.5061/dryad.x3ffbg7w0. Code (Saffarinia et al., 2025b) is available in Zenodo at https://doi.org/10.5281/zenodo.14503433. Raw abundance time‐series data can be accessed via their collecting agency, the California Department of Fish and Wildlife (https://wildlife.ca.gov/Conservation/Delta/Bay-Study; https://filelib.wildlife.ca.gov/Public/BayStudy/).
