# Supplementary material for: Time‐varying flow–ecology relationships for an endangered fish population: Longfin Smelt in the San Francisco Estuary
Source: Ecol Appl. 2026 Jan 19;36(1):e70178. doi: 10.1002/eap.70178 (PMC12813966; doi:10.1002/eap.70178)
Supplement: Supplementary file 1 — Appendix S1. [file EAP-36-e70178-s001.pdf]

Appendix S1

Time-varying flow–ecology relationships for an endangered fish population: Longfin Smelt in the San Francisco Estuary

Parsa Saffarinia, James A. Hobbs, Stephanie M. Carlson, Albert Ruhí

Ecological Applications

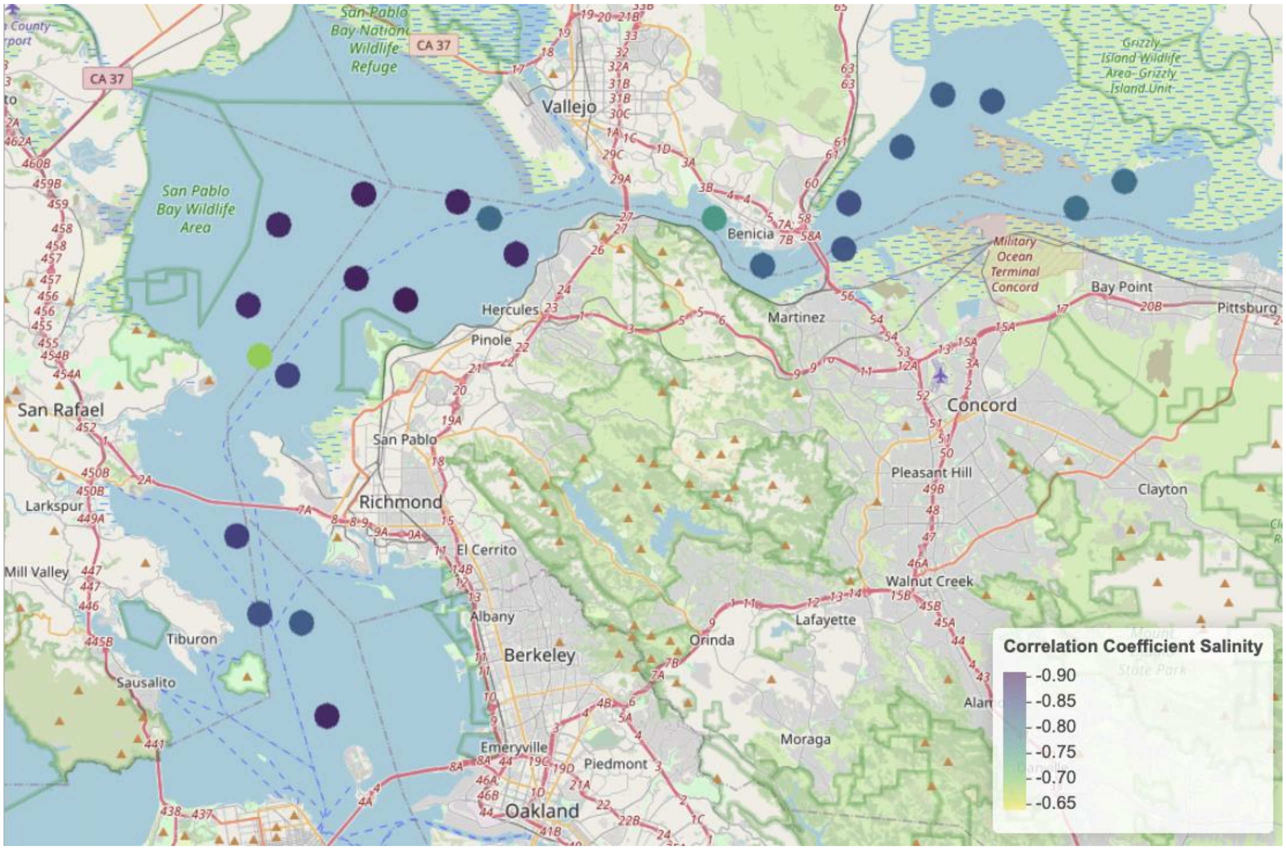

Figure S1. Pearson correlation coefficients between empirical, station-specific salinity values from the Bay Study dataset, and the outflow values used in our MARSS and DLM modeling exercises. The negative coefficients reflect the effects of freshening events (high Delta ouflows) on local salinity levels across the Estuary.

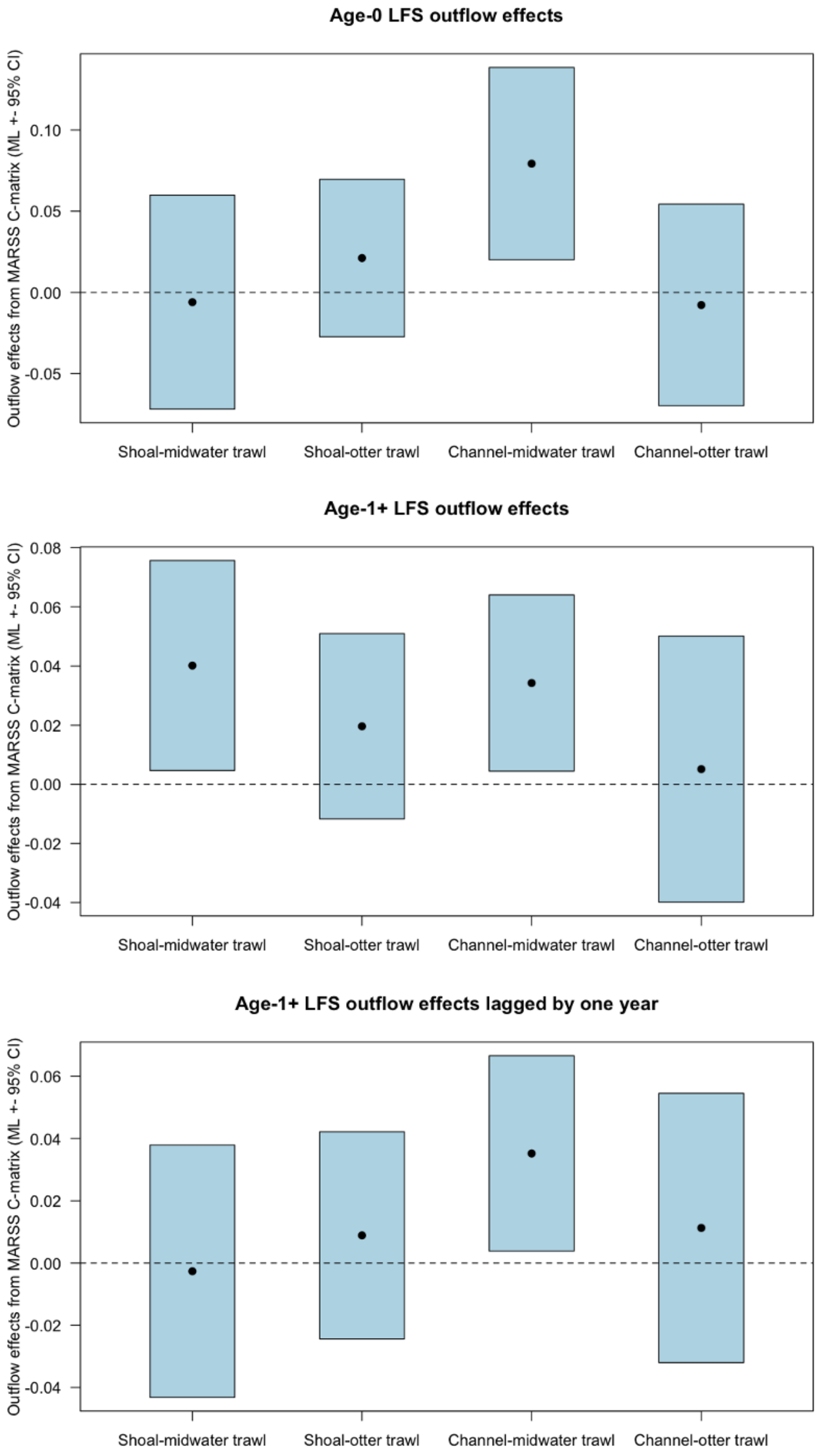

Figure 2. See next page for caption.

Figure S2. Outflow effects from MARSS models with a covariate (i.e., C matrix), estimated for each population group after determining the optimal spatial population structure. Black dots represent the maximum-likelihood effect, and the blue box represents 95% confidence intervals around the most likely effect. Each plot shows flow effects for an age class (age-0, age-1+) and flow lag (contemporary flow *vs.* 1-year lagged flow. Confidence intervals crossing y=0 (plotted as a dashed line) are not significantly different from zero.

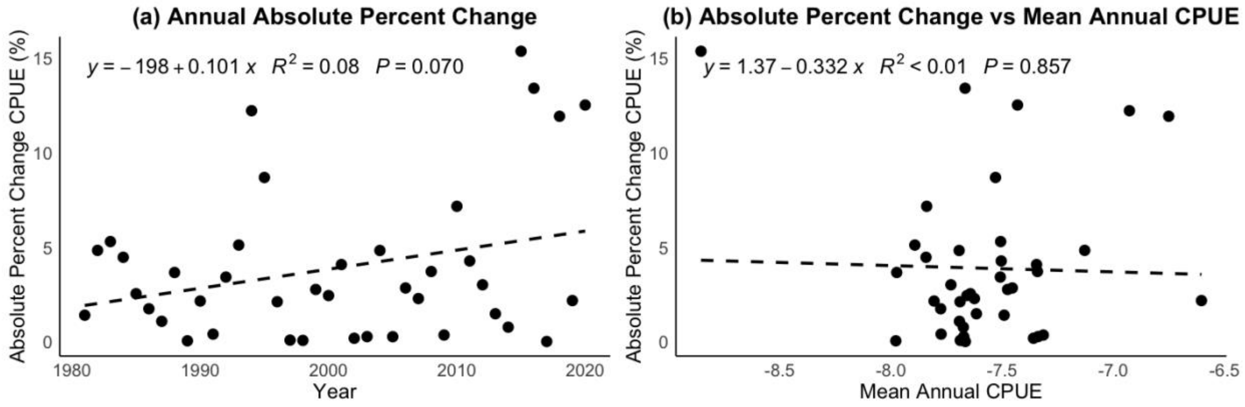

Figure S3. Annual variability in the mean catch-per-unit-effort (CPUE) of Longfin Smelt. *(a)* Annual absolute percent change in CPUE over time, demonstrating whether the magnitude of yearly variability has increased or decreased across years. *(b)* Relationship between mean annual CPUE and absolute percent change, highlighting whether higher CPUE values are associated with greater year-to-year variability. Both panels include regression lines along with equations indicating slope, R^2, and statistical significance (p-value).

Table S1. Station-specific population trajectories of Longfin Smelt, as described by long-term trends (Theil-Sen slopes) and variability (coefficients of variation, CV) of annual CPUE data. CV's were calculated as the ratio between the standard deviation and the mean of each time series, and are expressed as a percentage.

Station number	Habitat and gear type	Theil-Sen slope	Theil-Sen P-value	Theil-Sen Intercept	CV (%)
211	shoal midwater trawl	-2.11E-05	0.948	-8.62	32.4
211	shoal otter trawl	0.00248	0.403	-11.9	84.3
215	channel midwater trawl	-0.0121	0.00249	15.6	96.2
215	channel otter trawl	0.0123	0.00328	-31.5	120
216	channel midwater trawl	-0.0284	1.04E-05	48.4	269
216	channel otter trawl	0.00282	0.537	-12.2	86
317	shoal midwater trawl	0.011	0.218	-30.6	166
317	shoal otter trawl	0.0143	0.165	-35.4	76
318	shoal midwater trawl	0.00114	0.804	-11	85.7
318	shoal otter trawl	-0.0014	0.862	-4.06	110
319	shoal midwater trawl	0.00101	0.551	-11	92.9
319	shoal otter trawl	-0.00878	0.28	10.7	165
320	shoal midwater trawl	-0.00237	0.552	-4.08	89.9
320	shoal otter trawl	0.00164	0.563	-10.2	153
321	shoal midwater trawl	-0.0142	0.119	19.7	101
321	shoal otter trawl	0.00374	0.639	-14.3	180
322	shoal midwater trawl	-0.00279	0.643	-3.29	129
322	shoal otter trawl	-0.00348	0.597	0.0527	81.1
323	shoal midwater trawl	-0.00369	0.268	-1.33	245
323	shoal otter trawl	-0.00634	0.225	5.89	148
325	channel midwater trawl	-0.0163	0.00422	24	110
325	channel otter trawl	0.00634	0.301	-19.5	190
427	channel midwater trawl	-0.0094	0.00806	10.3	72.9
427	channel otter trawl	0.00696	0.158	-20.8	78.3
428	channel midwater trawl	-0.0177	0.000807	26.8	113
428	channel otter trawl	0.0271	0.0106	-60.6	173
429	channel midwater trawl	-0.00933	0.00482	10	118
429	channel otter trawl	-0.0133	0.0107	19.7	103

430	shoal midwater trawl	0.00614	0.401	-21.1	134
430	shoal otter trawl	-0.00209	0.467	-2.81	39.6
431	shoal midwater trawl	0.000202	0.881	-9.28	84.2
431	shoal otter trawl	0.00748	0.02	-21.9	41.5
432	channel midwater trawl	0.00574	0.385	-20	149
432	channel otter trawl	0.0114	0.0478	-29.4	99.7
433	channel midwater trawl	-0.0134	6.29E-05	18.2	96.1
433	channel otter trawl	0.00752	0.0745	-21.8	46.9
534	shoal midwater trawl	-0.000599	0.808	-7.65	31
534	shoal otter trawl	0.00605	0.052	-19.2	48.2
736	channel midwater trawl	-0.00259	0.526	-3.52	52.5
736	channel otter trawl	0.00486	0.332	-16.7	31.9
243	shoal midwater trawl	0.00415	0.0532	-17	183
243	shoal otter trawl	0.0121	0.0232	-31.2	221
345	channel midwater trawl	-0.00786	0.132	7.14	48.8
345	channel otter trawl	-0.0162	0.193	25.9	107
346	channel midwater trawl	0.00333	0.44	-15.2	70.7
346	channel otter trawl	0.00615	0.144	-19.1	65.1
447	shoal midwater trawl	-0.000461	0.0493	-8.08	21.9
447	shoal otter trawl	0.00813	0.000501	-23.3	32

Table S2. Mean population trajectories of Longfin Smelt collected by each habitat-gear type, as described by long-term trends (Theil-Sen slopes) and variability (coefficients of variation, CV) of annual CPUE data. See Figure 4 and Table S1 for station-specific data.

**Habitat and gear type**	**Mean Theil-Sen trend**	**Mean CV (%)**
Channel – Midwater trawl	-0.00982	109
Channel – Otter trawl	0.00509	100
Shoal – Midwater trawl	-3.76E-05	107
Shoal – Otter trawl	0.0026	106
